# Chromatin architecture reorganization during neuronal cell differentiation in *Drosophila* genome

**DOI:** 10.1101/gr.246710.118

**Published:** 2019-04

**Authors:** Keerthi T. Chathoth, Nicolae Radu Zabet

**Affiliations:** School of Biological Sciences, University of Essex, Colchester, CO4 3SQ, United Kingdom

## Abstract

The organization of the genome into topologically associating domains (TADs) was shown to have a regulatory role in development and cellular function, but the mechanism involved in TAD establishment is still unclear. Here, we present the first high-resolution contact map of *Drosophila* neuronal cells (BG3) and identify different classes of TADs by comparing this to genome organization in embryonic cells (Kc167). We find that only some TADs are conserved in both cell lines, whereas the rest are cell-type–specific. This is supported by a change in the enrichment of architectural proteins at TAD borders, with BEAF-32 present in embryonic cells and CTCF in neuronal cells. Furthermore, we observe strong divergent transcription, together with RNA Polymerase II occupancy and an increase in DNA accessibility at the TAD borders. TAD borders that are specific to neuronal cells are enriched in enhancers controlled by neuronal-specific transcription factors. Our results suggest that TADs are dynamic across developmental stages and reflect the interplay between insulators, transcriptional states, and enhancer activities.

Chromosome conformation capture Hi-C techniques have paved the way to dissecting the compartmental organization of genomes in various cell types (Dekker et al. 2002; Lieberman-Aiden et al. 2009; Dixon et al. 2012, 2015; Nora et al. 2012; Flyamer et al. 2017). Further advancements in high-resolution methodologies, such as in situ Hi-C, have enabled researchers to obtain much more refined 3D organization of the genome, from megabase-scale compartments to subkilobase resolution (Rao et al. 2014; Nagano et al. 2015; Cubeñas-Potts et al. 2017). Topologically associating domains (TADs) have been regarded as an important basic unit of chromosome organization (Dixon et al. 2012; Nora et al. 2012; Sexton et al. 2012). They are believed to be evolutionarily conserved and appear preserved across different organisms and cell types (Rao et al. 2014; Dixon et al. 2015; Vietri Rudan et al. 2015).

The majority of focused interactions observed within and between TADs, even those containing promoters at one end, are with regions devoid of any regulatory annotation. This suggests that TADs are not always regulatory in nature (Sanyal et al. 2012; Javierre et al. 2016). Nevertheless, there are also focused interactions that arise from enhancer–promoter interactions (Noordermeer et al. 2014; Cubeñas-Potts et al. 2017). Such dynamic regulation of long-range contacts (which is required for cell differentiation) is thought to occur within TADs. Similarly, the establishment of enhancer–promoter loops was shown to be tightly coupled to the activation of poised enhancers, as well as to gene expression (Freire-Pritchett et al. 2017). These internal interactions within TADs appear to change during development (Dixon et al. 2015) and under heat shock (Li et al. 2015). Although the functional importance of TADs was shown previously (Lupiañez et al. 2015), the factors contributing to stability and establishment of borders are not yet fully understood.

TADs are reported to be regions with low levels of active chromatin marks, which are separated by relatively high level of active marks (Ulianov et al. 2016; El-Sharnouby et al. 2017). Nevertheless, reports on reduced active marks within TADs are disputed, given the presence of enhancer–promoter loops within TADs (Noordermeer et al. 2014; Cubeñas-Potts et al. 2017). TAD borders were shown to be enriched with housekeeping and developmental enhancers (Cubeñas-Potts et al. 2017). The borders were also shown to coincide with long-range gene regulatory modules, such as genomic regulatory blocks (Harmston et al. 2017). Architectural proteins are considered to be another factor that plays a significant role in demarcating the TAD borders, and their enrichment has been correlated with border strength (Van Bortle et al. 2014; Stadler et al. 2017). CTCF and cohesin are the main architectural proteins that occupy mammalian TAD borders. The absence of these architectural proteins seems to disrupt TADs architecture unevenly, suggesting there are different types of borders (Zuin et al. 2014; Nora et al. 2017; Schwarzer et al. 2017). In contrast, TAD borders in *Drosophila* are occupied by a large set of insulator proteins, including CTCF, BEAF-32, Chromator (Chro), Cp190, etc. (Van Bortle et al. 2014; Stadler et al. 2017). Recently, transcription is emerging as another major driver of TAD formation (Li et al. 2015; Rowley et al. 2017). A recent study showed that TADs appear together with transcription activation in the zygote, but blocking transcription elongation does not seem to affect TADs (Hug et al. 2017). Synthetic induction of transcription using CRISPR/Cas9 system in mouse neuronal progenitor cells does not induce TAD border formation (Bonev et al. 2017).

Here, we aimed to understand the factors involved in TAD border formation in *Drosophila* and performed high-resolution in situ Hi-C experiments in *Drosophila* neuronal and embryonic cells that enabled high-resolution accurate demarcation of TAD borders. We used this new data set to provide new insights into the cell-type–specific borders that are gained or lost upon differentiation and also the interplay between enhancers and promoters, divergent transcription, and insulator proteins on TAD border formation in *Drosophila*.

## Results

### Characterization of TADs based on border conservation in *Drosophila*

**Figure 1.**
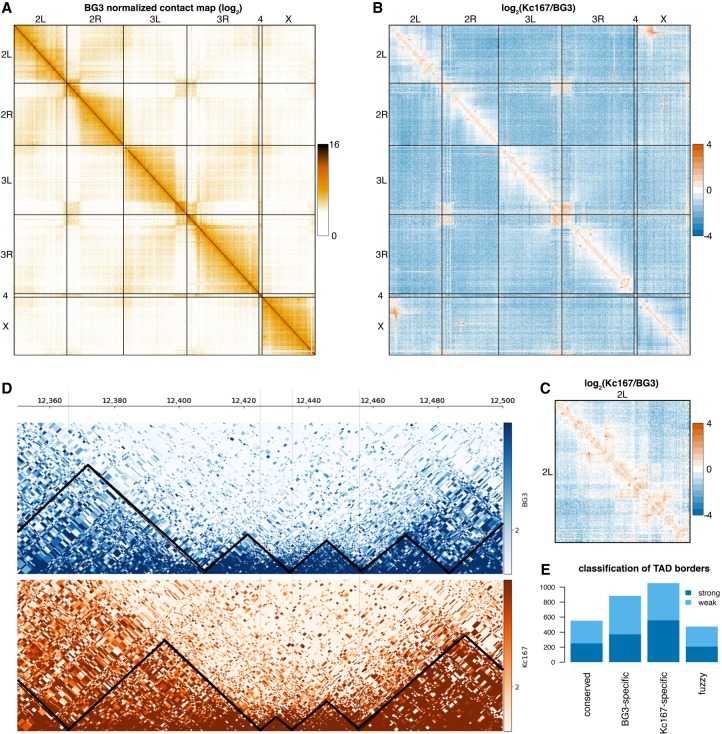
A high-resolution contact map of *Drosophila* BG3 cells. (*A*) Genome-wide normalized contact map of the *Drosophila* BG3 cell line at 100-kb resolution. Each element in the matrix represents the log_2_ of the normalized number of contacts between the two corresponding bins. (*B*) The log_2_ ratio between the normalized number of contacts in BG3 cells and Kc167 cells as indicated. (*C*) The log_2_ ratio between the normalized number of contacts in BG3 cells and Kc167 cells on Chromosome 2L. (*D*) Triangle view of the normalized contact map in BG3 cells at 2L:12,350,000–12,500,000 locus. Black lines demarcate the TADs. (*E*) Classification of TAD borders in BG3 cells as described in the main text: conserved borders, BG3-specific borders, Kc167-specific borders, and fuzzy borders. Depending on whether the TAD borders can still be detected when increasing the stringency of the TAD calling algorithm, we split each class of TAD border into two subgroups: strong borders and weak borders.

TADs have been analyzed in *Drosophila* previously (Hou et al. 2012; Sexton et al. 2012; Li et al. 2015; Ulianov et al. 2016; Cubeñas-Potts et al. 2017; Eagen et al. 2017; Hug et al. 2017; Rowley et al. 2017) using both low- and high-resolution approaches. All the previous in situ Hi-C studies (generating subkilobase-resolution contact maps) were conducted in embryonic cell lines (Kc167 and S2) or whole embryos (Cubeñas-Potts et al. 2017; Eagen et al. 2017; Hug et al. 2017; Rowley et al. 2017; Ramirez et al. 2018; Wang et al. 2018). Here we generated high-resolution chromatin maps of embryonic and neuronal cells in *Drosophila*, by performing in situ Hi-C in both Kc167 and BG3 cells (Fig. 1A). To generate this map, we used a four-base cutter (DpnII), which resulted in an average distance between restriction sites of ∼500 bp (see Methods). To understand the characteristics of chromatin organization in neuronal cells, we compared in situ Hi-C in BG3 cells with Kc167 (Fig. 1B,C). We used HiCExplorer (Ramirez et al. 2018) to analyze the in situ Hi-C data and identified 1909 TADs in BG3 cells and 2079 TADs in Kc167 cells (see Methods), which is in agreement with other studies (Cubeñas-Potts et al. 2017). This is almost four times more TADs in our Hi-C map of BG3 cells than previously reported from a low-resolution map (Ulianov et al. 2016). Compared with Kc167 cells, BG3 showed substantially higher long-range contacts and fewer short-range contacts, (Fig. 1B,C; Supplemental Fig. S1A–C). This suggests a dynamic change in chromatin and gene regulation upon differentiation.

During TAD border analysis, one-third of the borders appeared to be positioned at identical DpnII restriction sites between the two cell types, whereas the rest were positioned in varying distances (Supplemental Fig. S1G,H). Based on the conservation of the TAD borders in both cell types, we classified TAD borders as (1) BG3-specific (borders in BG3 cells that are at least 2 kb apart from the closest TAD border in Kc167 cells), (2) conserved (borders conserved at the exact DpnII restriction site in both Kc167 and BG3 cells), (3) Kc167-specific (borders in Kc167 cells that are at least 2 kb apart from the closest TAD border in BG3 cells), or (4) fuzzy (borders in BG3 cells that are slightly shifted within 2 kb of the corresponding border in Kc167 cells); see Figure 1, D and E. Depending on whether the TAD borders are still detected when increasing the stringency of the TAD calling algorithm, each class of TAD borders is further categorized into two subgroups: strong borders and weak borders (see Methods). To identify the possible strong determinants of establishment and maintenance of TAD borders, we focused on strong TAD borders in the first three classes identified in Figure 1E for our downstream analysis.

### Divergent transcription and polymerase occupancy associates with TAD borders

**Figure 2.**
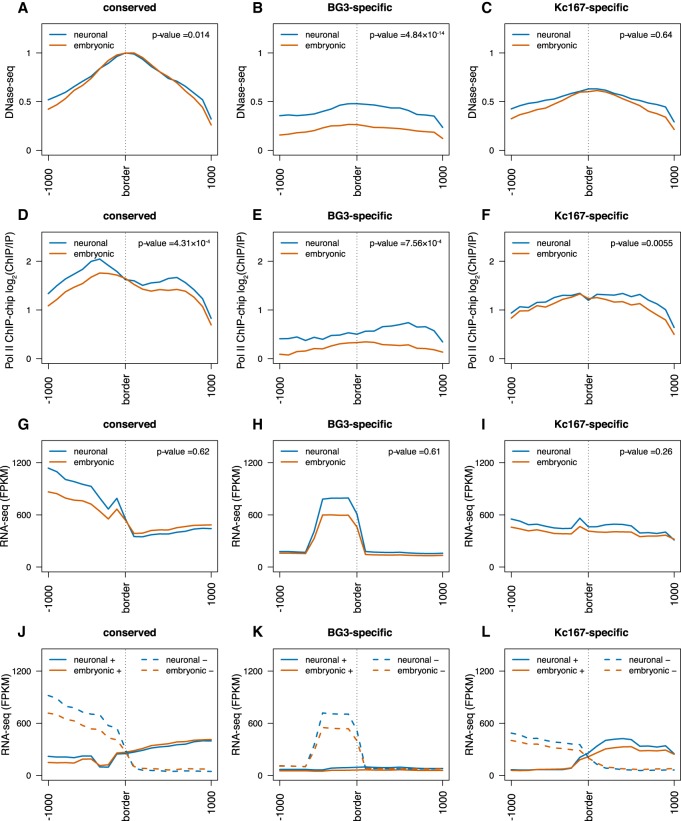
Divergent transcription and polymerase occupancy correlates with appearance of TAD borders. (*A–C*) DNase-seq signal at three different TAD border classes (conserved, BG3-specific, and Kc167-specific) as indicated. The red line represents data from embryonic cells (Kc167); the blue, from neuronal-derived cells (BG3). Average profile has been plotted considering 1 kb around each border. We performed a nonparametric Mann–Whitney *U* test considering the highest levels at each TAD border between embryonic and neuronal cells (see *P*-values). (*D–F*) Average Pol II ChIP-chip signal (log_2_ ChIP/input) at the borders of the three different TAD classes as indicated. (*G–I*) Average RNA-seq levels at the borders of the three different TAD classes as indicated. (*J–L*) Strand-specific average RNA-seq signal at the borders of the three different TAD classes showing strong divergent transcription at TAD borders. Solid lines represent the expression levels on the positive strand; dashed lines, the expression levels on the negative strand. For that, we mapped the expression levels of the genes to the corresponding strand using the FlyBase annotation (see Methods) (dos Santos et al. 2015).

Previous work showed that TAD borders display high levels of DNA accessibility and expression (Sexton et al. 2012; Li et al. 2015), but the role of active transcription as a determinant of TAD borders is still disputed (Bonev et al. 2017; Hug et al. 2017; Rowley et al. 2017). The expression of genes was shown to be a major predictor of TAD borders (Rowley et al. 2017). Nevertheless, blocking transcription with α-amanitin treatment does not remove TAD borders (Hug et al. 2017), whereas synthetic induction of genes with CRISPR/Cas9 system does not lead to the appearance of new TAD borders (Bonev et al. 2017). To analyze the transcriptional status between different cell types, we first compared the DNA accessibility and RNA polymerase II (Pol II) occupancy across different classes of TAD borders. DNA accessibility and Pol II occupancy were enriched at conserved borders and increased in BG3 cells at the BG3-specific borders (Fig. 2A–F). Nevertheless, at Kc167-specific borders, the enrichment of both DNA accessibility and Pol II occupancy was similar in Kc167 and BG3 cells. This suggests that relatively high DNA accessibility and Pol II occupancy are required for the establishment and maintenance of TAD borders but not sufficient.

Looking into the poly(A) RNA levels, in both embryonic and neuronal cells, we found that despite the increased presence of Pol II, total RNA expression showed negligible changes between the two cell lines at conserved or cell-type–specific borders (Fig. 2G–I). The little difference between the two cell types indicates that transcription alone cannot explain the appearance of TAD borders in BG3 cells, which is in agreement with previous studies (Bonev et al. 2017; Hug et al. 2017). As Pol II occupancy did not seem to completely explain the total RNA expression, we next analyzed the data in a strand-specific manner. A strong divergent transcription was present at all borders (Fig. 2J–L). To check if this phenomenon is encoded in the DNA sequence, we investigated the number of annotated genes at TAD borders and observed that the number of genes present at the TAD borders can explain the observed divergent transcription (Supplemental Fig. S2A–C). However, one exception was the right arm of the BG3-specific TAD borders (Fig. 2K). To account for this, we reasoned that this part of divergent transcription is contributed by ncRNAs, as the number of encoded ncRNAs appears to be highly enriched on the positive strand at these loci (Supplemental Fig. S2D–F). To elucidate this, we then examined the nascent RNA in both cell lines (Supplemental Fig. S2G–L). As a result, we observed divergent transcription at BG3-specific borders in BG3 cells (Supplemental Fig. S2J–L), which indicates a potential role for ncRNA in sustaining divergent transcription and, consequently, in the formation or maintenance of TAD borders. However, the presence of divergent transcription observed in BG3 cells at Kc167-specific borders (Supplemental Fig. S2J–L) indicates that divergent transcription may be required for the establishment and maintenance of TAD borders, but probably additional factors are also needed.

**Figure 3.**
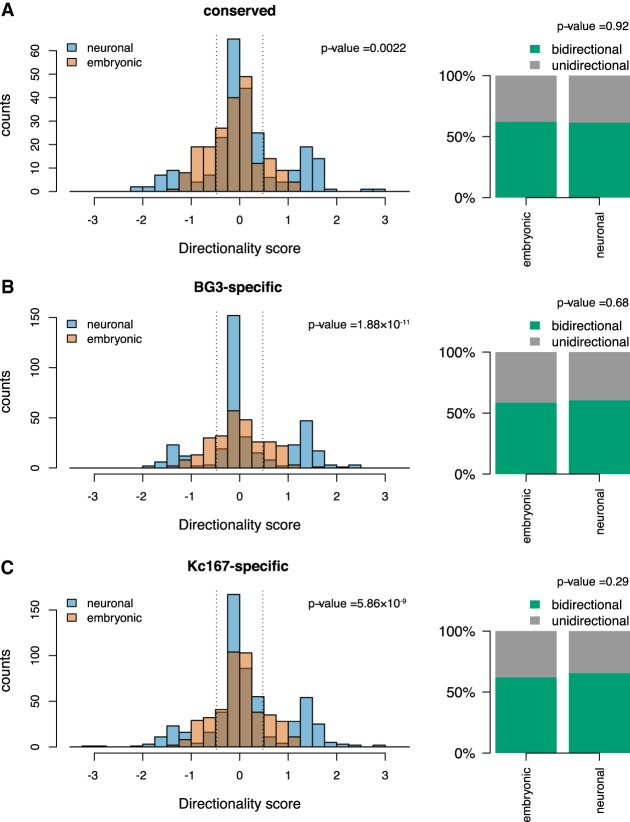
Bidirectional transcription at TAD borders. Histograms representing the directionality score computed as log_10_ of the ratio between nascent RNA levels in 500 bp on the positive strand downstream from the border and on the negative strand upstream of the border; 500-bp bins that were 500 bp away were considered in both directions from the border. The barplot represents the percentage of TAD borders in which the directionality score was lower than 0.47 (dotted lines on the histogram, representing less than three times more transcription on one strand). We classified these borders as bidirectional borders. We used a Kolmogorov–Smirnov test to compare the change in distribution of bidirectionality score (see *P*-values) and a Fisher's exact test to compare if the change in number of bidirectional TAD borders is statistically significant (see *P*-values). (*A*) Conserved TAD borders; (*B*) BG3-specific TAD borders; and (*C*) Kc167-specific TAD borders.

To further validate the different aspects of divergent transcription, we looked at the ratio between sense and antisense transcription. Our results show that there is more antisense transcription on the left side of TAD borders and more sense transcription on the right side of TAD borders (Supplemental Fig. S3A–F). Finally, by using the definition of divergent transcription as less than three times more nascent transcription on one strand compared with the other strand in 500-bp windows (Jin et al. 2017), we observed that the majority of TAD borders display bidirectional transcription (Fig. 3). There is a high percentage of borders with bidirectional transcription in all cases (Fig. 3A–C). Altogether, our data show that increased Pol II occupancy and divergent transcription are associated with TAD borders, but additional factors are needed to drive their formation.

### Architectural proteins at borders show differential occupancy between cell lines with CTCF being enriched in BG3 cells

**Figure 4.**
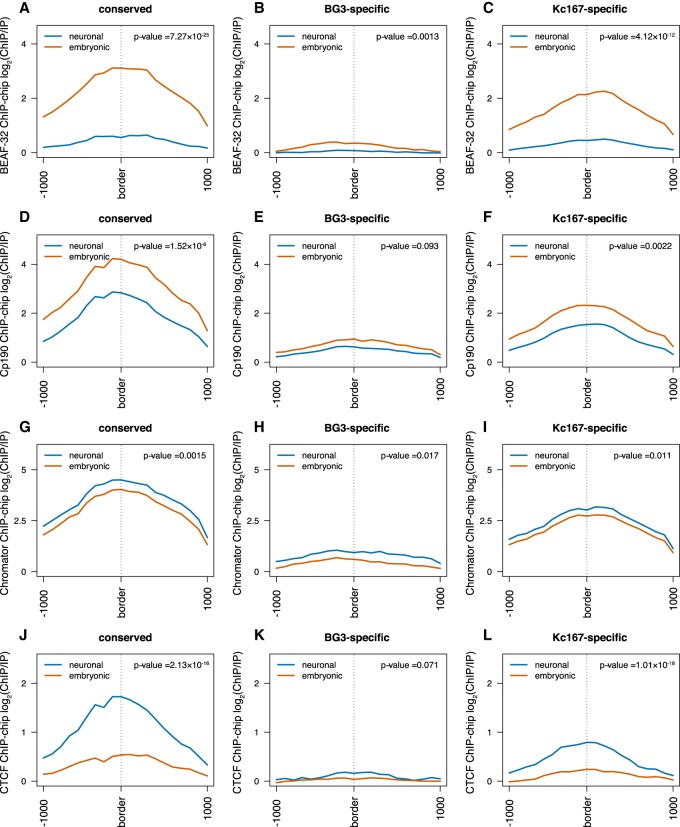
Architectural proteins differentially occupy TAD borders in a cell-type–specific manner. (*A–C*) Average BEAF-32 ChIP-chip signal (log_2_ ChIP/input) at three different TAD border classes (conserved, BG3-specific, and Kc167-specific). The red line represents data from embryonic cells; the blue, from neuronal-derived cells. As before, 1 kb around each border was considered while plotting the average profile. We performed a nonparametric Mann–Whitney *U* test considering the highest levels at each TAD border between embryonic and neuronal cells (see *P*-values). (*D–F*) Average Cp190 ChIP-chip signal (log_2_ ChIP/input) at the borders of the three different TAD classes. (*G–I*) Average Chromator ChIP-chip signal (log_2_ ChIP/input) at the borders of the three different TAD classes. (*J–L*) Average CTCF ChIP-chip signal (log_2_ ChIP/input) at the borders of the three different TAD classes.

*Drosophila* displays a large repertoire of architectural proteins at TAD borders (Van Bortle et al. 2014). BEAF-32, Cp190, and Chromator are involved in long-range interactions (Vogelmann et al. 2014), and several recent studies also found they are the most enriched proteins at TAD borders in *Drosophila* (Cubeñas-Potts et al. 2017; El-Sharnouby et al. 2017; Hug et al. 2017; Ramirez et al. 2018; Wang et al. 2018). We also observed that the insulator proteins mentioned above were enriched especially at the conserved borders (Fig. 4). When comparing the two cell lines, BEAF-32 and Cp190 are present at higher levels in embryonic cells than in neuronal cells especially at conserved and Kc167-specific borders (Fig. 4A–F), whereas the Chromator signal is slightly enriched at TAD borders of both cell lines (Fig. 4G–I).

In contrast to mammalian systems, previous studies reported reduced amounts of CTCF binding at TAD borders in *Drosophila*. However, those studies were performed either in embryonic cells (Ramirez et al. 2018; Wang et al. 2018) or by use of low-resolution Hi-C data sets in differentiated cells (Ulianov et al. 2016). We found that CTCF binds more at TAD borders than previously reported, and this increase in binding is substantially higher in BG3 cells at conserved borders (Fig. 4J–L). CTCF was shown to promote long-range interactions in *Drosophila* (Li et al. 2013), as well as to regulate developmentally stable interactions (Phillips-Cremins et al. 2013). Given that long-range contacts are more prevalent in neuronal cells (Bonev et al. 2017), an increased presence of CTCF and Chromator in *Drosophila* may mediate such interactions either independently or in combination with other architectural proteins. Apparently, Kc167-specific borders show low levels for both BEAF-32 and CTCF in BG3 cells (Fig. 4C,L). Putting these results together indicates that the loss of Kc167-specific borders in BG3 cells could be explained by the loss of binding of BEAF-32 in BG3 cells from these borders, which is not compensated by CTCF. In contrast, at conserved borders, the loss of BEAF-32 in BG3 cells is compensated by a stronger recruitment of CTCF, which may explain the maintenance of these borders.

We also examined other architectural proteins such as Su(Hw) and Trl (Supplemental Fig. S4). The Trl profile was similar to Chromator across the border and between cell types, with slight specific enrichment at BG3-specific borders in BG3 cells (Supplemental Fig. S4A–C). Consistent with previous reports, Su(Hw) is less enriched at TAD borders (Supplemental Fig. S4D–F; Ulianov et al. 2016; Ramirez et al. 2018). The differential enrichment of certain architectural proteins may indicate their importance in TAD border establishment and gene regulation during different developmental stages, potentially leading to the formation and maintenance of cell-type–specific chromatin organization states.

### BG3-specific TAD borders are enriched with active enhancer marks

Previous studies reported the presence of histone marks associated with active transcription (H3K27ac, H3K4me1, and H3K4me3) at TAD borders and the presence of repressive marks associated with silent genes inside TADs (H3K27me3) (Ulianov et al. 2016; Cubeñas-Potts et al. 2017; El-Sharnouby et al. 2017). Here, at BG3-specific borders, BG3 cells displayed an increased signal for H3K27ac (active enhancers) and H3K27me3 (repressive mark) (Supplemental Fig. S5). Note that although H3K27ac displays a peak around the TAD border, H3K27me3 displays a valley around the same TAD borders. The presence of both active and repressive marks indicate that BG3-specific TAD borders are in a bivalent chromatin state in BG3 cells (enhancer regions that contain both repressive and activating marks) (Skalska et al. 2015). At Kc167-specific borders, Kc167 cells display intermediary levels of H3K27ac and low levels of H3K27me3, whereas BG3 cells show similar H3K27ac levels and increased levels of H3K27me3 (Supplemental Fig. S5C,F). This suggests significant accumulation of repressive marks at Kc167-specific borders after differentiation, which could explain their loss in BG3 cells.

Looking at other histone marks, we found that conserved TAD borders show low levels of H3K4me1 and an enrichment for H3K4me3 in both cell lines, indicating that these borders are associated with actively transcribed genes (or housekeeping genes) (Supplemental Fig. S5G–L; Skalska et al. 2015). At Kc167-specific borders, Kc167 cells display reduced H3K4me1 levels that do not change after differentiation (statistically insignificant) (Supplemental Fig. S5I) and enhanced H3K4me3 levels (similarly to conserved borders) that are lost after differentiation. Again, this supports a model in which Kc167-specifc borders are enriched in genes that are down-regulated after differentiation. In contrast, BG3-specific TAD borders display slightly higher levels of H3K4me1 and are depleted of H3K4me3 in BG3 cells. This shows that BG3-specific borders are enriched in developmental enhancers but are not associated with active promoters (Supplemental Fig. S5; Skalska et al. 2015). Altogether, above results propose that conserved TAD borders are enriched in constitutively expressed genes, BG3-specific TAD borders are enriched in BG3-specific enhancers, and Kc167-specific borders are enriched in genes that are down-regulated after differentiation.

### Enhancers are pronounced at the borders during differentiation in *Drosophila*

**Figure 5.**
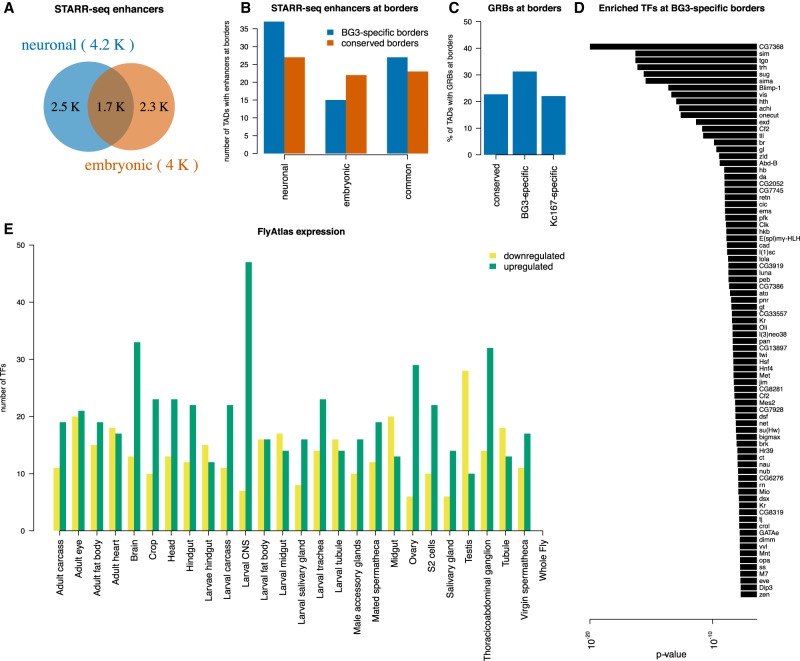
Cell-type–specific enhancers correlate with cell-type–specific TAD borders. (*A*) Venn diagram representing the number of enhancers in neuronal and embryonic *Drosophila* cells as identified by STARR-seq. Enhancers were classified as cell specific if they were annotated only in one cell type or as common if they were annotated in both cell types. (*B*) The number of conserved (red) or BG3-specific (blue) TAD borders that overlap with a cell-type–specific or common enhancer. Barplot showing more BG3-specific borders with neuronal enhancers than with common or embryonic enhancers, unlike conserved TAD borders. (*C*) The percentage of TAD borders that overlap with gene regulatory blocks (GRBs) (Harmston et al. 2017). A higher number of BG3-specific borders overlap with GRBs compared with conserved borders (Fisher's exact test *P*-value = 0.022) or Kc167-specific borders (Fisher's exact test *P*-value = 0.0021), but there is no difference between conserved borders and Kc167-specific borders (Fisher's exact test *P*-value = 0.86). (*D*) List of 81 TFs with enriched motifs at BG3-specific borders and the associated *P*-value (see Methods). (*E*) Expression of 79 of these TFs in different tissues/cells from FlyAtlas data set. Green represents up-regulated genes; yellow, down-regulated genes as indicated.

Enhancer–promoter looping is a contributing factor to gene regulation and is considered to be one of the factors that possibly drives TAD formation (Bonev and Cavalli 2016). These enhancer–promoter interactions are subjected to changes during differentiation depending on the transcriptional requirement of the cells (Ghavi-Helm et al. 2014). TAD borders were previously classified as being established either by housekeeping genes or by developmental enhancers in embryo-derived cells (Kc167) (Cubeñas-Potts et al. 2017). Here, we provide further evidence for this classification of TAD borders by investigating if the TAD borders that are specific to BG3 cells are enriched in neuronal-associated enhancers compared with TAD borders that are conserved between two cells. We used the STARR-seq data (Arnold et al. 2013; Yanez-Cuna et al. 2014) to classify enhancers as BG3-specific, S2-specific (*Drosophila* embryonic cell line), and common enhancers (Fig. 5A). We found more BG3-specific TAD borders that contain neuronal enhancers than BG3-specific TAD borders that contain common or embryonic enhancers (Fig. 5B). In contrast, the number of conserved TAD borders containing BG3-specific (neuronal) or S2-specific (embryonic) enhancers was similar. This supports the model that enhancer–promoter looping may be one of the underlying factors for BG3-specific TAD border formation. We also looked into the clusters of noncoding regulatory elements called gene regulatory blocks (GRBs) (Harmston et al. 2017) at the TAD borders. We found increased number of GRBs coinciding at BG3-specific borders compared with conserved or Kc167-specific ones (Fig. 5C).

Nevertheless, despite the increase of neuronal-specific enhancers and GRBs at BG3-specific borders, a large proportion of BG3 borders was still depleted of enhancers or GRBs. This was puzzling because we observed active transcription and enhancer marks at these TAD borders (Fig. 2; Supplemental Fig. S5). Thus, we used a complementary approach in which we identified a list of 81 transcription factors that have their binding motifs enriched at BG3-specific TAD borders (see Methods) (Fig. 5D). By using the FlyAtlas data set (Chintapalli et al. 2007), we checked if these transcription factors are expressed and specific to any particular tissue/cell, and we found expression data for 79 of them. Notably, a majority of the 79 transcription factors that have motifs enriched at BG3-specific TAD borders is specifically expressed in the larval central nervous system (from where BG3 cells were derived) and brain (Fig. 5E). This provides additional evidence that BG3-specific enhancers contribute to the formation of BG3-specific TAD borders.

### BG3 shows more long-range contacts, and Kc167 shows more short-range interaction

**Figure 6.**
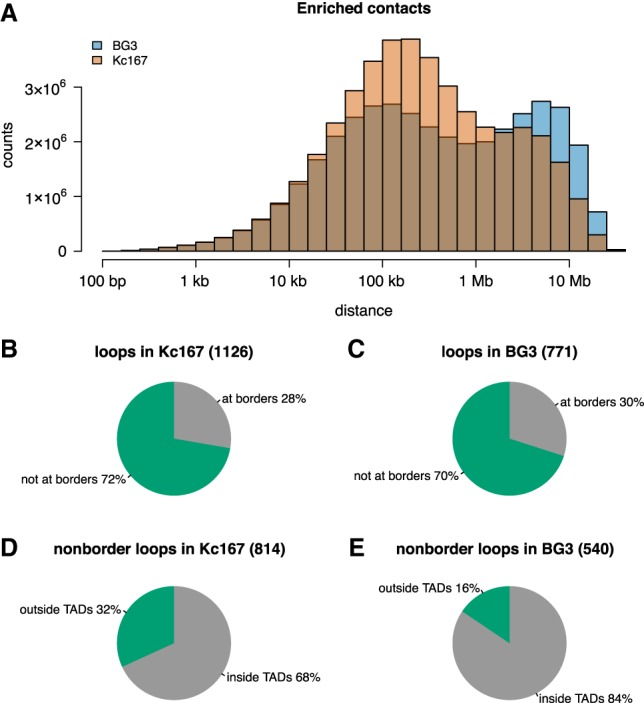
BG3 cells display more long-range interactions compared with Kc167 cells. (*A*) Histogram showing the distances between two anchors of enriched contacts in the contact matrices (for BG3 and Kc167 cells). To keep consistency, we considered the down-sampled Kc167 Hi-C map, which had the same number of interactions as the BG3 map. (*B*,*C*) Distribution of chromatin loops at the borders of TADs in Kc167 and BG3 cells, showing that both cells have similar number of loops at TAD borders (Fisher's exact test *P*-value = 0.3). (*D*,*E*) Distribution of nonborder chromatin loops within TADs or between TADs in Kc167 and BG3 cells, showing that Kc167 cells display more loops within TADs compared with BG3 cells (Fisher's exact test *P*-value = 6.61 × 10^−12^).

It was reported that *Drosophila* displays significantly fewer chromatin loops than found in mammals (Eagen et al. 2017). Our first inspection of the Hi-C map (Fig. 1B) indicated that BG3 cells would have more long-range contacts, whereas Kc167 would display more short-range contacts. To analyze long- and short-range interactions in detail, we identified all enriched contacts in the Hi-C data set in BG3 and Kc167 cells. As expected, we observed more enriched contacts in Kc167 cells at distances between 10 kb and 1 Mb and more enriched contacts in BG3 cells for distances >1 Mb (Fig. 6A).

Next, we detected chromatin loops in the two cell types using HiCCUPS (Durand et al. 2017) and identified that there are more loops in Kc167 (1126) than BG3 (771) cells. Approximately one-third of the loops in both Kc167 and BG3 cells are located at TAD borders (Fig. 6B,C). However, we observed that there are more loops that are inside TADs in BG3 compared with Kc167 (Fig. 6D,E). This suggests that, in BG3 cells, there are fewer TADs, but these are larger and they contain more loops inside the TADs.

Finally, almost half of the loops in both cells have a promoter at one anchor, independent of whether the loop is inside TADs or between different TADs; whereas 10% of the loops have an ncRNA annotated at one anchor (Supplemental Fig. S6). This indicates that active transcription can be involved in maintaining these loops, by bridging either enhancers to promoters or promoters to promoters, in order to coordinate gene expression (by the location of genes in transcription factories). In contrast, we found that only a small percentage of loops (10%–20%) have an enhancer annotated at one anchor. However, improvements in techniques or analysis tools (Eagen et al. 2017; Muerdter et al. 2018) may likely increase the number of enhancer–promoter loops identified.

### A/B compartment switching is not connected with TAD borders changes

**Figure 7.**
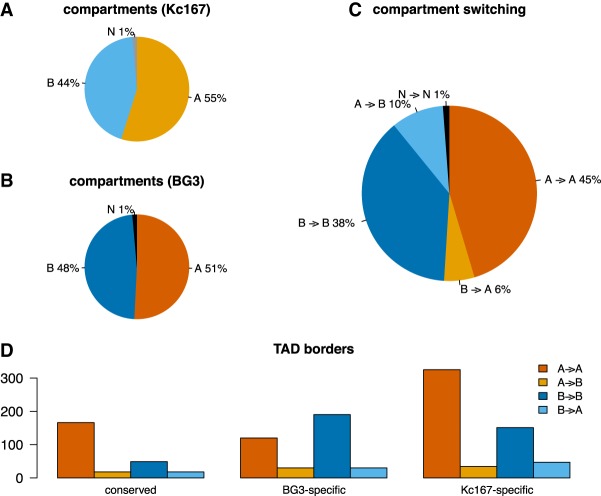
A/B compartments in Kc167 and BG3 cells. (*A,B*) Percentage of the genome that was computed as either an A or a B compartment (Lieberman-Aiden et al. 2009). Regions that could not be classified as either an A or a B compartment were labeled as N. (*C*) The percentage of A/B compartments that switched after differentiation from embryonic to neuronal cells. (*D*) TAD borders location within the A/B compartments.

Eukaryotic genomes are portioned in A/B compartments, where A marks active regions of chromatin and B marks inactive regions of chromatin (Lieberman-Aiden et al. 2009). Previous work has identified the presence of A/B compartments in *Drosophila* (Rowley et al. 2017), and following a similar approach, we computed the A/B compartments in both Kc167 and BG3 cells at 10-kb-bin resolution (see Methods). Our results show that there is an increase of B compartments in BG3 cells, with more A compartments switching to B (10%), compared with B compartments switching to A (6%) (Fig. 7A–C). We did not identify any correlation between the compartment switching or the gain or loss of TAD borders in BG3 cells compared with Kc167 (Fig. 7D). However, we observed a preference of both conserved and Kc167-specific TAD borders for A compartments in contrast to a preference of BG3-specific borders for B compartments (Fig. 7D).

## Discussion

There have been several reports recently regarding TADs in *Drosophila* (Cubeñas-Potts et al. 2017; Eagen et al. 2017; Hug et al. 2017; Rowley et al. 2017; Ramirez et al. 2018; Wang et al. 2018). All of these studies generated high-resolution Hi-C maps in embryo-derived cells or whole embryos. We present here, for the first time, an in situ Hi-C map of a differentiated neuronal cell line (BG3) in *Drosophila*, which enabled us to study different aspects of chromatin reorganization during *Drosophila* differentiation.

A large proportion of TADs appear conserved across various cell types, but there are also TADs that are cell specific and dictate cellular identity. Here, with the high-resolution data sets in BG3, we were able to identify TAD borders accurately and compare them with TAD borders in Kc167 cells (embryo-derived). Thus, both conserved and new TAD borders gained in BG3 cells were identified (Fig. 1E). TADs are regarded as mostly conserved between different cell types (Dixon et al. 2012; Vietri Rudan et al. 2015). We show that, in addition to common TADs, the two different cell types from the same organism also display different TADs. Varying transcriptional status, and enhancer–promoter interactions across different developmental stages of cells, may impact the reorganization of underlying functional elements. This reorganization can, in turn, influence the 3D structure of the genome.

### Role of transcription and DNA-binding proteins on TAD border formation

Transcription was considered a major factor for TAD formation (Li et al. 2015; Rowley et al. 2017), whereas depletion of transcription did not seem to affect the TAD formation (Hug et al. 2017). However, the presence of concomitant divergent transcription and strong Pol II signals observed at the borders suggest a potential role for transcription or Pol II to be associated with TAD formation (Fig. 2).

Divergent transcription is enriched in mammalian systems and was assumed to be depleted in *Drosophila*, but recent reports showed that divergent transcription is more prominent in *Drosophila melanogaster* than previously estimated (Rennie et al. 2018). We observed divergent transcription at all borders, which indicates its association with TAD borders in *Drosophila*. How would the presence of divergent transcription lead to formation of TADs? One possibility is that divergent transcription produces negative supercoiling in the promoter region of the sense gene, which is maintained during transcription by polymerase, topoisomerase, and helicases (Naughton et al. 2013a,b). This is also probably one of the reasons why arresting polymerase by α-amanitin did not affect TAD structures (Hug et al. 2017) as α-amanitin only blocks the elongation of Pol II but does not displace Pol II from the DNA or change the supercoiling state of the DNA. Additionally, it was shown by numerical simulations using DNA polymer models, that divergent transcription–induced supercoiling could explain the self-interacting chromatin domains in *Schizosaccharomyces pombe* (Benedetti et al. 2017). This hypothesis could possibly be extended to other species like *Drosophila* and other higher eukaryotes in which divergent transcription could be involved in the establishment or maintenance of actual TAD structures.

Divergent transcription–induced supercoiling was proposed as a mechanism to release paused polymerase in *Drosophila* (revised in Naughton et al. 2013b). Trl and M1BP are associated with Pol II pausing (Li and Gilmour 2013), and Trl was shown previously to be involved in repressive chromatin loops within polycomb domains (Ogiyama et al. 2018). We observed specific enrichment of Trl at BG3 borders in BG3 cells (Supplemental Fig. S4). Also, it is worthwhile noting that the divergent transcription observed at TAD borders is owing to transcription not only of protein coding genes but also of ncRNA (Supplemental Fig. S2). This indicates that cell-type–specific ncRNAs could have a role in TAD border formation and maintenance and, consequently, in gene regulation. However, the presence of divergent transcription in BG3 cells at Kc167-specific borders suggests that, although divergent transcription is associated with TAD border formation, it is not enough to establish these borders after differentiation.

Architectural proteins are the other factors regarded to play a significant role in determining the TAD border formation (Van Bortle et al. 2014). Some of these proteins involved in long-range interactions (such as Chromator and CTCF) were enriched in BG3 cells, validating the presence of distal interactions observed in these cell lines. In this study, we show that TAD borders in *Drosophila* are enriched with CTCF in neuronal cells (which commonly occupies mammalian TAD borders and was less pronounced in *Drosophila* embryonic TAD borders) (Fig. 4). The higher occupancy of CTCF in BG3 cells can be explained by higher amounts of CTCF in this cell line (Zabet and Adryan 2015). In combination with Chromator and Cp190, they can mediate long-range interactions to meet the transcriptional requirement of the cell during neuronal differentiation. After differentiation, Kc167-specific borders lose binding of BEAF-32, which is not compensated by a strong recruitment of CTCF, as is the case for conserved borders. Despite their presence at the borders, not all architectural proteins were particularly enriched at the BG3-specific borders and therefore may not be essential for the border formation in BG3 cells (Supplemental Fig. S4).

### Role of enhancers on TAD border formation in BG3 cells

Binding of transcription factors to enhancers activates transcription of target genes (Calo and Wysocka 2013). Here, we showed that BG3-specific borders display active enhancer marks (Supplemental Fig. S5) and are enriched in binding motifs of transcription factors that are expressed specifically in neuronal cells (Fig. 5), thus supporting that the BG3-specific TAD borders are enriched in BG3-specific enhancers. In contrast, we found that Kc167-specific TAD borders are enriched in promoter marks (H3K4me3) and seem to display intermediate expression compared with the conserved TAD borders (Supplemental Fig. S5). This suggests that Kc167-specific TAD borders contain regulated genes that are expressed at intermediary levels but lose promoter marks after differentiation. Thus, the presence of active enhancer marks, along with the neuronal-specific enhancers and GRBs, at BG3-specific borders emphasize the role of enhancers in driving TAD border formation during differentiation.

Our data support a change in organization of the genome observed across different cell stages. Like in mammalian cells, CTCF might have a role in *Drosophila* that needs to be explored in more detail using different cell types. Furthermore, our results suggest that there is a dynamic change in the enhancer–promoter interaction, especially at BG3-specific borders. This may indicate a role for enhancer–promoter interaction as one of the contributing factors for TAD formation. Similar observations have been reported in human cells (Bonev et al. 2017; Freire-Pritchett et al. 2017), which suggests that the reorganization during development is conserved during evolution.

Finally, we hypothesize that active divergent transcription could generate negative supercoiling and, in turn, may facilitate the formation of compacted domains that are conserved through different developmental stages. The role of the architectural proteins would be to help maintain this negative supercoiling and keep the state of the system stable. However, further work is needed to elucidate the extended role of each factor in TAD formation.

## Methods

### Cell culture

*Drosophila* BG3 cells were cultured at 25°C in Schneider's Insect Medium (Sigma-Aldrich), supplemented with 10% FBS (Labtech), 10 mg/L insulin (Sigma-Aldrich I9278), and antibiotic penicillin/streptomycin.

### In situ Hi-C protocol

Hi-C libraries were generated from 10 million cells by following the in situ Hi-C protocol as mentioned by Rao et al. (2014), with minor modifications. Crosslinked cells were lysed, and the genome was digested using DpnII (NEB) overnight. The overhangs were filled with Biotin-16-dATP (Jena Bioscience) followed by ligation and decrosslinking with Proteinase K digestion. The sample was further sonicated using Bioruptor. Biotinylated DNA was pulled down using Dynabeads MyOne streptavidin T1 beads (Life Technologies 65602). Selected biotinylated DNA fragments ranging from 200–500 bp were then ligated with Illumina adaptors (NEB). The libraries obtained from biological replicates were multiplexed and further sequenced at Edinburgh Genomics (Genepool) and Fasteris using HiSeq 4000.

### Hi-C analysis

Each pair of the PE reads was aligned separately to the *D. melanogaster* (dm6) genome (Adams et al. 2000; dos Santos et al. 2015) using BWA-MEM (with options -t 20 -A1 -B4 -E50 -L0) (Li and Durbin 2010). HiCExplorer was used to build and correct the contact matrices and to detect TADs and enriched contacts (Ramirez et al. 2018). The contact matrices were built at 100-kb bins for plotting Figure 1 and at 10 kb for compartments (Rowley et al. 2017), and the DpnII restriction sites were used for calling TADs and chromatin loops and plotting the rest of the figures. By using a minimum allowed distance between restriction sites of 150 bp and a maximum distance of 1 kb, we obtained a matrix with 217,638 bins with a median width of 529 bp. After filtering, the two BG3 replicates had 40 million and 41 million reads, whereas the two Kc167 replicates had 48 million and 60 million reads. In addition, we down-sampled the Kc167 libraries (Kc167 subset), leading to a similar number of filtered reads as in the case of BG3 cells. We merged the two BG3 biological replicates and the two Kc167 replicates, which did not change the contacts decay curves (Supplemental Fig. S1A–C). The matrices were corrected using the thresholds (−1.4 and 5 for DpnII restriction sites and 10-kb bins, whereas for 100-kb bins, we used −2.4 and 5 for BG3 data and −3.0 and 5 for Kc167 data); values were selected from the diagnostic plots (Supplemental Fig. S1D–F). By using the corrected contact matrices, we detected TADs of at least 5-kb width using a *P*-value threshold of 0.01, a minimum threshold of the difference between the TAD-separation score of 0.04, and FDR correction for multiple testing (--step 2000, --minBoundaryDistance 5000 --pvalue 0.01 --delta 0.04 --correctForMultipleTesting fdr). We selected these parameters to ensure that we recovered a similar number of TADs in Kc167 cells as previously reported (Cubeñas-Potts et al. 2017). We compared the distances between TAD borders and found that using the full Kc167 libraries or the subset libraries would lead to similar results (Supplemental Fig. S1G,H). Finally, we called strong TAD borders using a stringent value of the threshold of the difference between the TAD separation score of 0.08. This value ensured that we retrieved the strongest half of TADs.

The enriched contacts were extracted with HiCExplorer using the observed/expected ratio method. In order for the two cells to be comparable, we used the Kc167 subset data set instead of the full data set. The same analysis was performed for both data sets generated in this study (in situ Hi-C of BG3 and Kc167 cells).

Because the Kc167 cell line is a female-derived cell line and BG3 is a male-derived cell line, we excluded sex chromosomes from our analysis in order to avoid any biases from dosage compensation (Mukherjee and Beermann 1965; Chiang and Kurnit 2003). The downstream analysis and plots were generated using a custom script in R (R Core Team 2017); see Supplemental Code.

### Motif enrichment analysis

The analysis to identify enriched motifs at TAD borders was performed with R/Bioconductor package PWMEnrich (http://bioconductor.org/packages/PWMEnrich/). First, we created a background model using the lognormal method, 200-bp sequence lengths, and all Kc167-specific TAD borders (all borders in Kc167 that were further by at least 2 kb from any TAD border in BG3). Enriched binding motifs that had a *P*-value lower than 0.05 were selected, which resulted in 81 TFs. Finally, by using FlyMine (Lyne et al. 2007), we extracted the FlyAtlas expression data for 79 of these TFs (Chintapalli et al. 2007).

### Chromatin loops

Chromatin loops were called with the HiCCUPS tool from the Juicer software suite on both Kc167 and BG3 data sets (Durand et al. 2017). Loops were called using a 2-kb resolution, 0.05 FDR, Knight–Ruiz normalization, a window of 10, peak width of five, thresholds for merging loops of 0.02,1.5,1.75,2, and distance to merge peaks of 20 kb (-k KR -r 2000 -f 0.05 -p 5 -i 10 -t 0.02,1.5,1.75,2 -d 20000). For details on the parameters, see the work of Durand et al. (2017).

### Compartments

Compartments were called as previously described (Lieberman-Aiden et al. 2009; Rowley et al. 2017). More specifically, we used Juicer to compute the eigenvectors in 10-kb bins for both the Kc167 and BG3 data sets (Durand et al. 2017). The sign of the correlation between the GC content and eigenvectors was used to flip the sign of the eigenvector (Imakaev et al. 2012). Bins with negative eigenvalues were assigned as a B compartment, whereas bins with positive eigenvalues were assigned as an A compartment.

### Data sets

Note that because of the similarities in chromatin nature and transcriptional profiles of the Kc167 and S2 cell types (Cubeñas-Potts et al. 2017), depending on data availability, we used one of the two as the embryonic cell line when comparing with the neuronal cell line BG3. To maintain consistency with our TAD annotation, if files had coordinates in other release versions of the *D. melanogaster* genome, the coordinates were lifted to dm6.

### ChIP-chip

We used the following ChIP-chip data sets generated and preprocessed (M values smoothed over 500 bp) by The modENCODE Consortium (Kharchenko et al. 2010; The modENCODE Consortium et al. 2010; Riddle et al. 2011; Schwartz et al. 2012): Pol II (GSE20832, GSE20806), BEAF-32 (GSE20811, GSE20760), Cp190 (GSE20814, GSE20766), Chromator (GSE20761, GSE20763), CTCF (GSE20767, GSE32818), Trl (GSE23466, GSE32822), Su(Hw) (GSE20833, GSE51964), H3K27ac (GSE20778, GSE20779), H3K27me3 (GSE20780, GSE45083), H3K4me1 (GSE23468, GSE45085), and H3K4me3 (GSE20839, GSE45088).

### RNA-seq

The mRNA abundance in the two cell lines was downloaded from the work of Lee et al. (2014), who preprocessed the original modENCODE data sets (Cherbas et al. 2011). To obtain the strand-specific expression, we mapped the genes on the corresponding strand using the FlyBase annotation (dos Santos et al. 2015). For nascent RNA transcription, we used the preprocessed GRO-seq in S2 cells (GSM577244) from the work of Core et al. (2012) and the preprocessed 3′NT-seq in BG3 cells (GSE100545) from the work of Pherson et al. (2017).

### Other data sets

We also used gene and ncRNA annotations for Supplemental Figure S2 from FlyBase (dos Santos et al. 2015), STARR-seq annotation of enhancers in BG3 and S2 cells from Arnold et al. (2013) and Yanez-Cuna et al. (2014), and preprocessed DNase-seq profiles from The modENCODE Consortium (Kharchenko et al. 2010).

### Data analysis

The scripts to perform the analysis are made available as Supplemental Code. For the Fisher's exact test (Figs. 3, 5C, 6B–E) we also performed a two-sample randomization (permutation) test to compare two proportions. The obtained *P*-values were identical as reported by the Fisher's exact test, except for Figure 5C at the difference between BG3-specific and Kc167-specific TAD borders that are overlapped with GRB in which the *P*-value was *P*-value = 0.0017 (instead of *P*-value = 0.0021 for the Fisher's exact test).

## Data access

All Hi-C data from this study have been submitted to the NCBI Gene Expression Omnibus (GEO; https://www.ncbi.nlm.nih.gov/geo/) under accession number GSE122603. The pipeline for Hi-C data analysis is available as Supplemental Code.

## Supplementary Material

Supplemental Material
