# Supplementary material for: Chromatin architecture reorganization during neuronal cell differentiation in Drosophila genome
Source: Genome Res. 2019 Apr;29(4):613–25. doi: 10.1101/gr.246710.118 (PMC6442379; doi:10.1101/gr.246710.118)
Supplement: Supplemental Material [file supp_29_4_613__index.html]

Chromatin architecture reorganization during neuronal cell differentiation in Drosophila genome — Supplemental Material 

# Chromatin architecture reorganization during neuronal cell differentiation in *Drosophila* genome

## Supplemental Material

- Supplemental\_Fig\_S1.pdf
- Supplemental\_Fig\_S2.pdf
- Supplemental\_Fig\_S3.pdf
- Supplemental\_Fig\_S4.pdf
- Supplemental\_Fig\_S5.pdf
- Supplemental\_Fig\_S6.pdf
- Supplemental\_code.zip
