# Supplementary material for: Chromatin architecture reorganization during neuronal cell differentiation in Drosophila genome
Source: Genome Res. 2019 Apr;29(4):613–25. doi: 10.1101/gr.246710.118 (PMC6442379; doi:10.1101/gr.246710.118)
Supplement: Supplemental Material [file supp_gr.246710.118_Supplemental_Fig_S1.pdf]

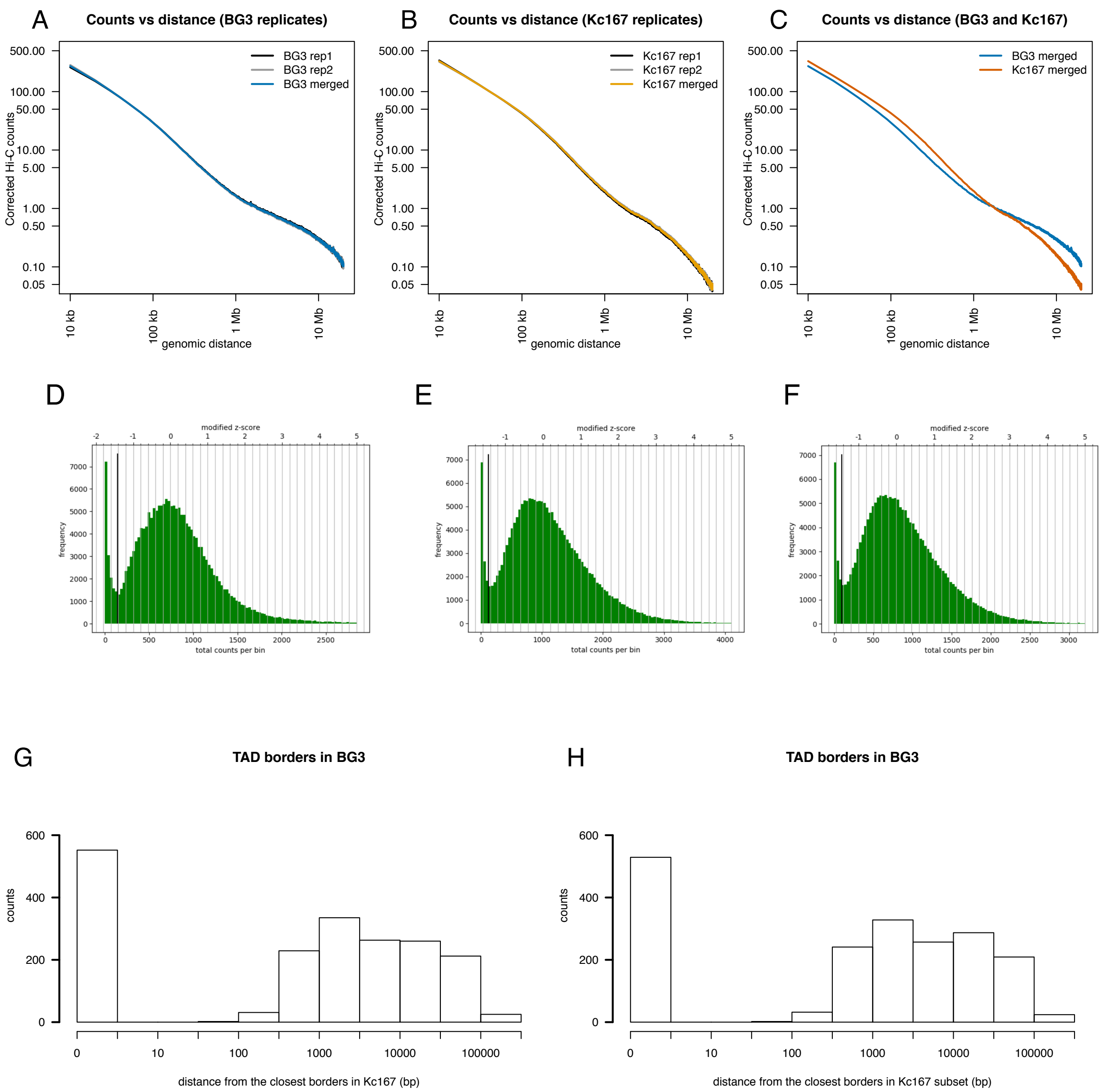

Fig S1. Conservation of TAD borders.
(A-C) Counts vs distance plots for the Hi-C datasets. (A) BG3 replicate 1, BG3 replicate 2 and BG3 merged. (B) Kc167 replicate 1, Kc167 replicate 2 and Kc167 merged. (C) BG3 merged and Kc167 merged. (D-F) Diagnostic plots for correction of Hi-C plots from HiCExplorer representing histograms of the sum of contact per bin in (D) BG3 cells, (E) Kc167 cells and (F) Kc167 subset. The vertical black line represents the lower threshold for removing bins with lower number of reads. (G-H) Histograms showing the distances between TAD borders in BG3 cells and the closest TAD border in (G) Kc167 and (H) Kc167 subset datasets.
