# Supplementary material for: Chromatin architecture reorganization during neuronal cell differentiation in Drosophila genome
Source: Genome Res. 2019 Apr;29(4):613–25. doi: 10.1101/gr.246710.118 (PMC6442379; doi:10.1101/gr.246710.118)
Supplement: Supplemental Material [file supp_gr.246710.118_Supplemental_Fig_S2.pdf]

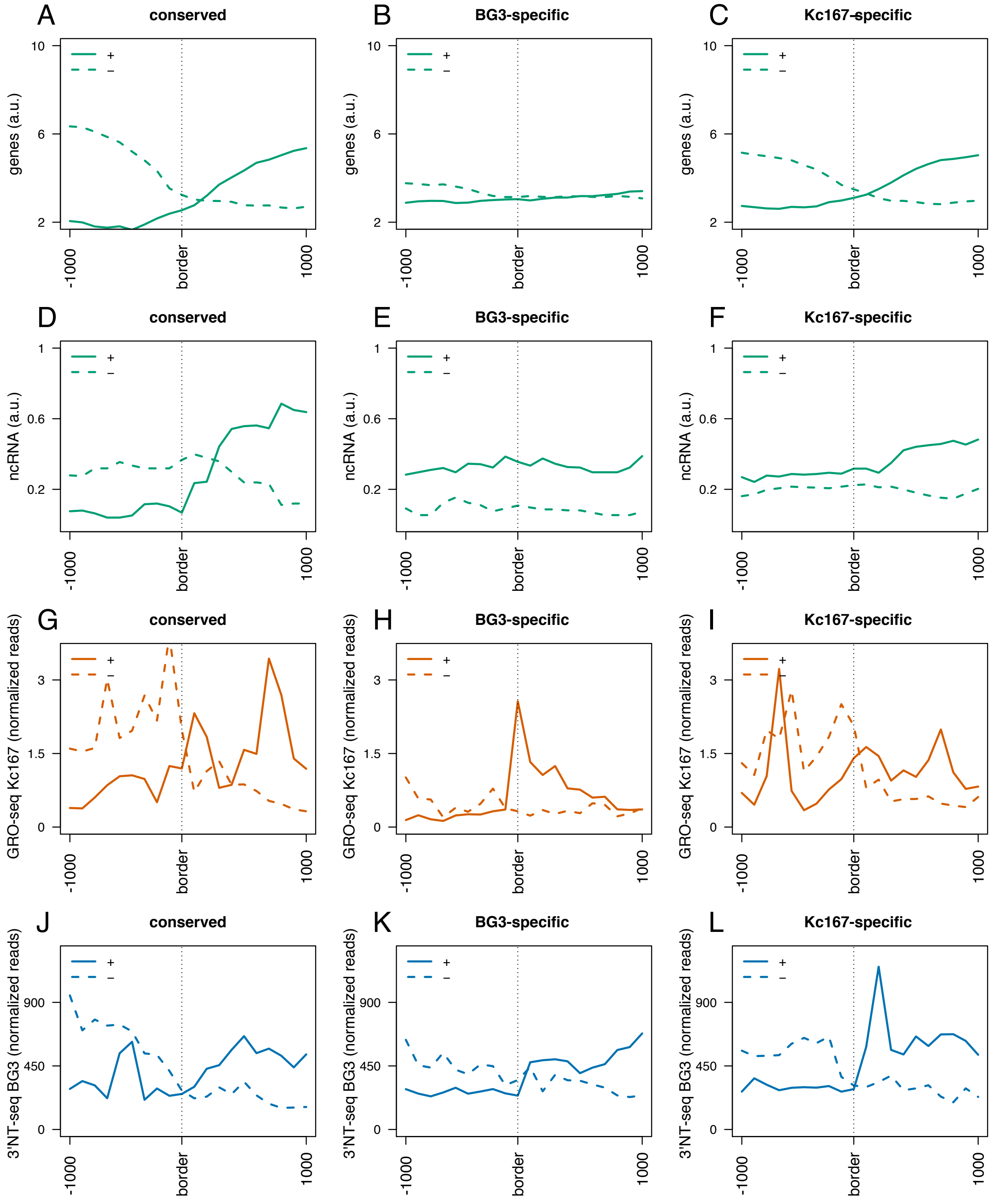

Figure S2. Divergent transcription at TAD borders.

(A-C) Average number of strand specific genes encoded around (A) conserved, (B) BG3-specific and (C) Kc167-specific TAD borders in *Drosophila*. Solid lines represent the positive strand and dashed lines the negative strand. (D-F) same as (A-C), for ncRNAs. (G-I) Average GRO-seq signal in embryonic cells (S2) around (G) conserved, (H) BG3-specific and (I) Kc167-specific TAD borders. (J-L) Average 3'NT-seq signal in neuronal cells (BG3) around (J) conserved, (K) BG3-specific and (L) Kc167-specific TAD bordersBG3-specific.
