# Supplementary material for: Chromatin architecture reorganization during neuronal cell differentiation in Drosophila genome
Source: Genome Res. 2019 Apr;29(4):613–25. doi: 10.1101/gr.246710.118 (PMC6442379; doi:10.1101/gr.246710.118)
Supplement: Supplemental Material [file supp_gr.246710.118_Supplemental_Fig_S3.pdf]

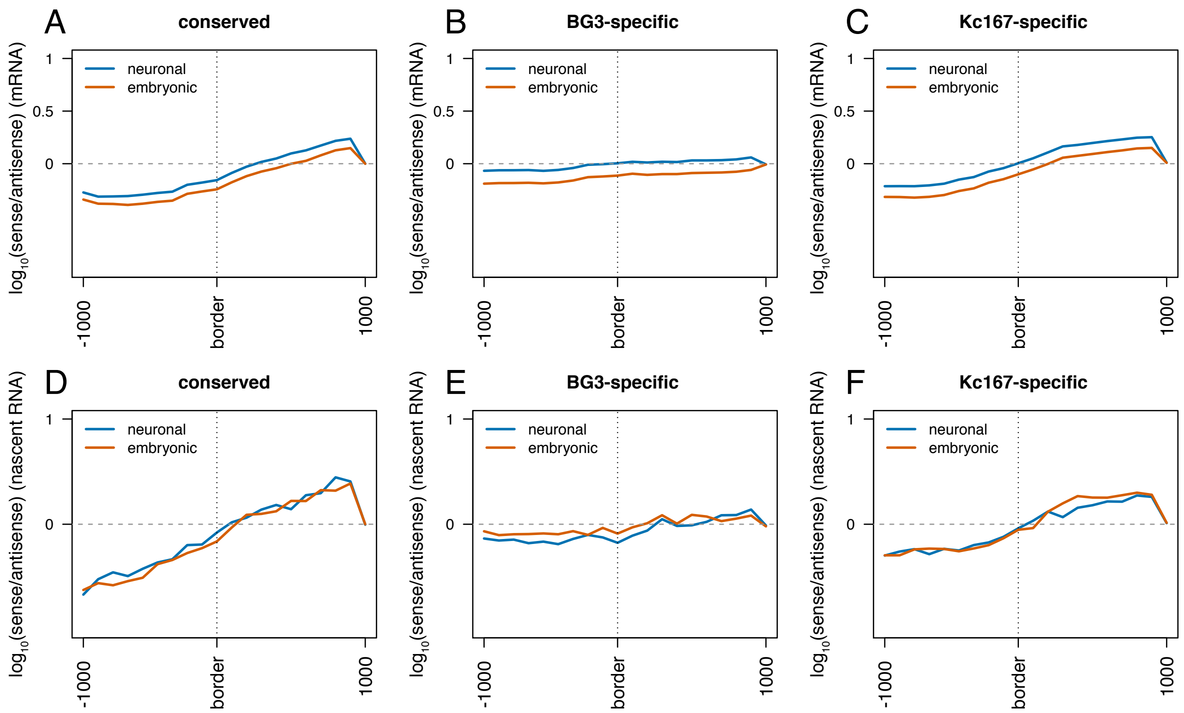

Figure S3. Sense and antisense transcription at TAD borders.
Average of the log_{10} ratio between sense and antisense transcription for embryonic cells and neuronal cells at (A and D) conserved, (B and E) BG3-specific and (C and F) Kc167-specific TAD borders. (A-C) total mRNA levels (D-F) nascent RNA (GRO-seq in S2 and 3'NT-seq in BG3 as mentioned in method section).
