# Supplementary material for: Chromatin architecture reorganization during neuronal cell differentiation in Drosophila genome
Source: Genome Res. 2019 Apr;29(4):613–25. doi: 10.1101/gr.246710.118 (PMC6442379; doi:10.1101/gr.246710.118)
Supplement: Supplemental Material [file supp_gr.246710.118_Supplemental_Fig_S4.pdf]

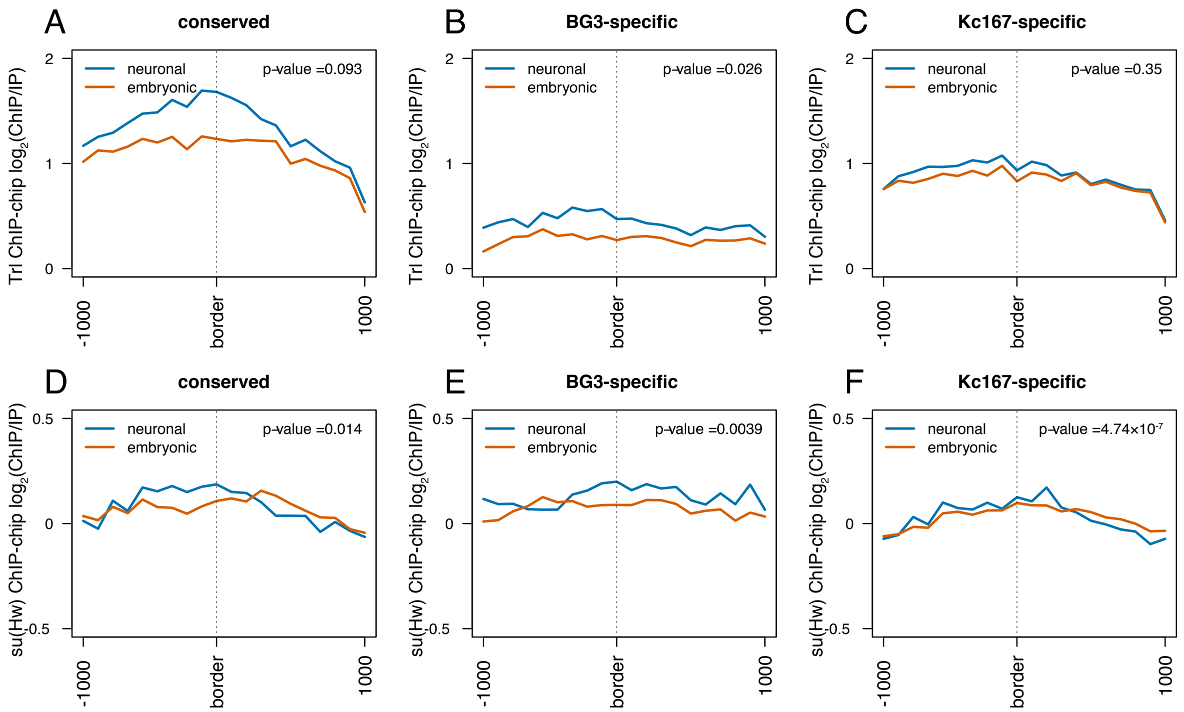

Figure S4. Only some architectural proteins differentially occupy TAD borders in a cell-specific manner.
(A-C) Average Trl ChIP-chip signal (log_2 ChIP/input) at borders of three different TAD classes (conserved, BG3-specific and Kc167-specific). Red line represents data from embryonic cells and blue from neuronal derived cells. Average profile is plotted as mentioned in Fig 4. We performed a non-parametric Mann-Whitney *U* test considering the highest levels at each TAD border between embryonic and neuronal cells (see reported p-value). (D-F) Average su(Hw) ChIP-chip signal (log_2 ChIP/input) at borders of the three different TAD classes.
