# Supplementary material for: Chromatin architecture reorganization during neuronal cell differentiation in Drosophila genome
Source: Genome Res. 2019 Apr;29(4):613–25. doi: 10.1101/gr.246710.118 (PMC6442379; doi:10.1101/gr.246710.118)
Supplement: Supplemental Material [file supp_gr.246710.118_Supplemental_Fig_S5.pdf]

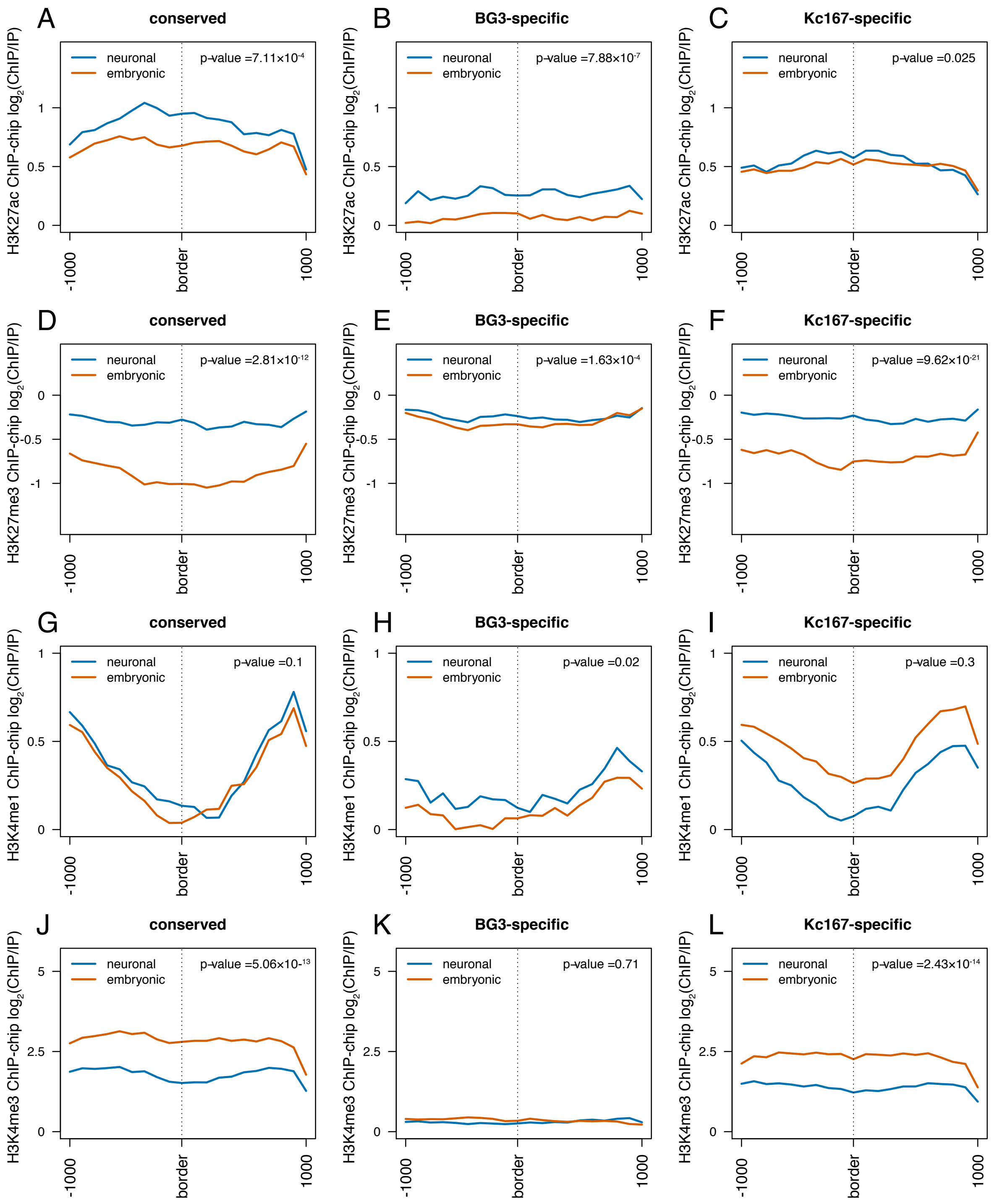

Figure S5. Histone marks are present at TAD borders in a cell-specific manner. Average levels of histone marks, ChIP-chip signal (log_2 ChIP/input), at borders of three different TAD classes (conserved, BG3-specific and Kc167-specific). Red line represents data from embryonic cells and blue from neuronal derived cells. Average profile is plotted considering 1 kb around each border. We performed a non-parametric Mann-Whitney *U* test considering the highest levels at each TAD border between embryonic and neuronal cells (see reported p-value). We plotted the following histone marks: (A-C) H3K27ac (D-F) H3K27me3 (G-I) H3K4me1 and (J-L) H3K4me3.
