# Supplementary material for: Chromatin architecture reorganization during neuronal cell differentiation in Drosophila genome
Source: Genome Res. 2019 Apr;29(4):613–25. doi: 10.1101/gr.246710.118 (PMC6442379; doi:10.1101/gr.246710.118)
Supplement: Supplemental Material [file supp_gr.246710.118_Supplemental_Fig_S6.pdf]

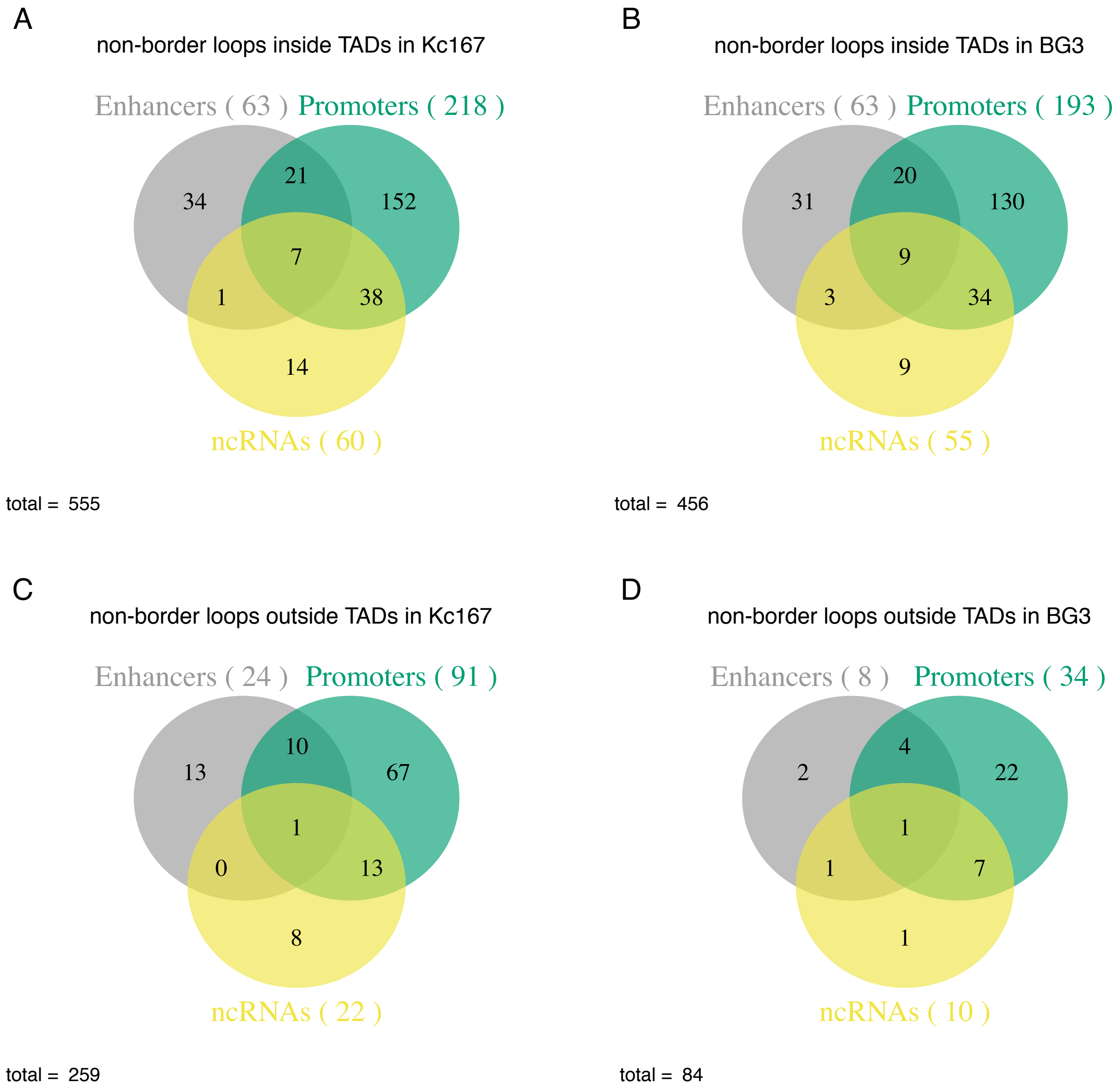

Figure S6. Annotations of the anchors of chromatin loops.
Venn diagrams representing the number of chromatin loops that have at one of the anchors promoters (green), ncRNAs (yellow) or enhancers (grey). (A-B) Chromatin loops where both anchors are inside the same TAD in (A) Kc167 cells and (B) BG3 cells. (C-D) Chromatin loops where the anchors are in different TADs in (C) Kc167 cells and (D) BG3 cells.
